# Validation of the electronic Holistic Needs Assessment

**DOI:** 10.1186/s40064-015-1401-0

**Published:** 2015-10-19

**Authors:** Austyn Snowden, Mick Fleming

**Affiliations:** School of Nursing Midwifery and Social Care, Edinburgh Napier University, Edinburgh, Scotland, UK; School of Health Nursing and Midwifery, University of the West of Scotland, Paisley, Scotland, UK

**Keywords:** Cancer, Holistic needs assessment, Validity, Reliability, Rasch analysis, Factor analysis

## Abstract

Macmillan Cancer Support UK have developed an electronic Holistic Needs Assessment (eHNA) to: (1) help people living with cancer express all their needs, (2) help those helping them better target support. eHNA consists of 48 items each ranked from zero (no problem) to 10. There has been no psychometric analysis of this tool and so its validity and reliability are untested. The aim of this study was to evaluate the psychometric properties of the eHNA by examining its construct validity. Objectives were to (a) test whether the eHNA measured holistic concerns and (b) analyse the factor structure of the eHNA. Objectives were achieved through a secondary analysis of 5421 responses to eHNA using concurrent application of Rasch analysis and principal component analysis. All the items bar one fit with the Rasch rating model and were equivalently important to people. Differential item functioning was evident according to whether people were described as curative or not. A 12-factor solution explained 46 % variance. Of this the emotional/spiritual factor explained the most variance accounting for 15 %. The eHNA was internally consistent and conceptually coherent with the construct of holistic needs assessment. Clinical focus is best directed to the individual items highlighted by the patient except where patients check too many problems for the clinician to accurately prioritise. In these cases only, the emotional/spiritual factor may help identify appropriate clinical action. Strengths and weaknesses of the analyses are discussed, particularly in relation to ‘at risk’ subsamples such as those classified as non-curative.

## Background

Many cancer survivors have moderate to severe unmet needs, often as consequences of treatment (Carey et al. 2012). Changing the way cancer survivors are supported remains an ongoing priority (Tavernier 2014). Much of this work has focused on better understanding and acting on people’s individual holistic needs (Rooney et al. 2014), and various tools and strategies have been developed to facilitate this agenda (Henry et al. 2014). Initially the most widely used tool was the distress thermometer (DT), developed primarily to help assess the psychological needs of people affected by cancer (Holland and Bultz 2007). However the DT was found to be limited in relation to articulating all the relevant holistic needs of people affected by cancer (Mitchell et al. 2010). The need for a different tool emerged from these perceived shortcomings (Snowden and White 2014).

Holistic needs assessment aims to:

- Highlight the unmet needs of people affected by cancer.
- Enable healthcare professionals to focus on those needs in a structured way.
- Enable appropriate services to meet those needs.
- Aid the development of an individualised care plan (National Cancer Action Team 2013).

The electronic version of holistic needs assessment (eHNA) is constructed from 48 items grouped within five domains: physical, practical, social, emotional and spiritual. Each item is scored from zero (no problem) to 10 (maximum concern). The checklist is completed by the person with cancer on a tablet PC and the results discussed with a relevant professional. Despite very positive anecdotal feedback from patients and services the tool has not been psychometrically tested. This study is the first to assess its construct validity.

Construct validity concerns the degree to which a test measures what it is supposed to measure. In this case the eHNA is supposed to be measuring concerns of people with cancer. It is also designed to be a holistic measure. Every item should therefore represent a ‘concern’, be equivalently important, and provide unique information. Unusually then, instead of looking for a factor structure or redundant items to reduce the item bank, this study is essentially looking for an *absence* of a factor structure and no redundant items in order to confirm the construct validity of the eHNA.

## Aim

The aim of this study is to evaluate the psychometric properties of the eHNA by examining its construct validity.

### Objectives

1. Test the degree to which the eHNA measures holistic concerns.
2. Assess the factor structure of the eHNA.

## Design

Secondary analysis of an existing dataset of responses to eHNA using concurrent application of rasch analysis and exploratory factor analysis.

### Participants

The sample consisted of 5421 people with cancer (1923 males, 3497 females, 1 not reported) with mean (SD) age 62.7 (14.6) years. All completed eHNA in south England 2014–2015. In total, people reported 34,656 problems. Mean (SD) number of problems reported was 6.39 (5.86) with a range of 0–47 problems identified. Mean total eHNA score (max potential range 0–480) was 30.5 (33) with a range of 366. Mean time for completion was 7.33 (6.33) minutes with a range of 58 min. There was a significant but small correlation between number of problems reported and length of time for completion (r = 0.186, p < 0.001). The correlation was slightly higher when total eHNA score was correlated against time (r = 0.266, p < 0.001).

**Fig. 1:**
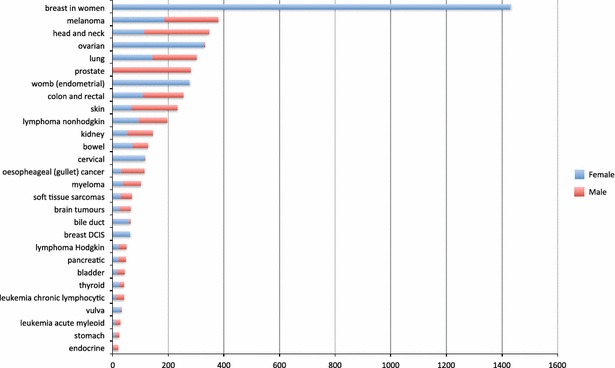
Diagnosis ranked by frequency

**Fig. 2:**
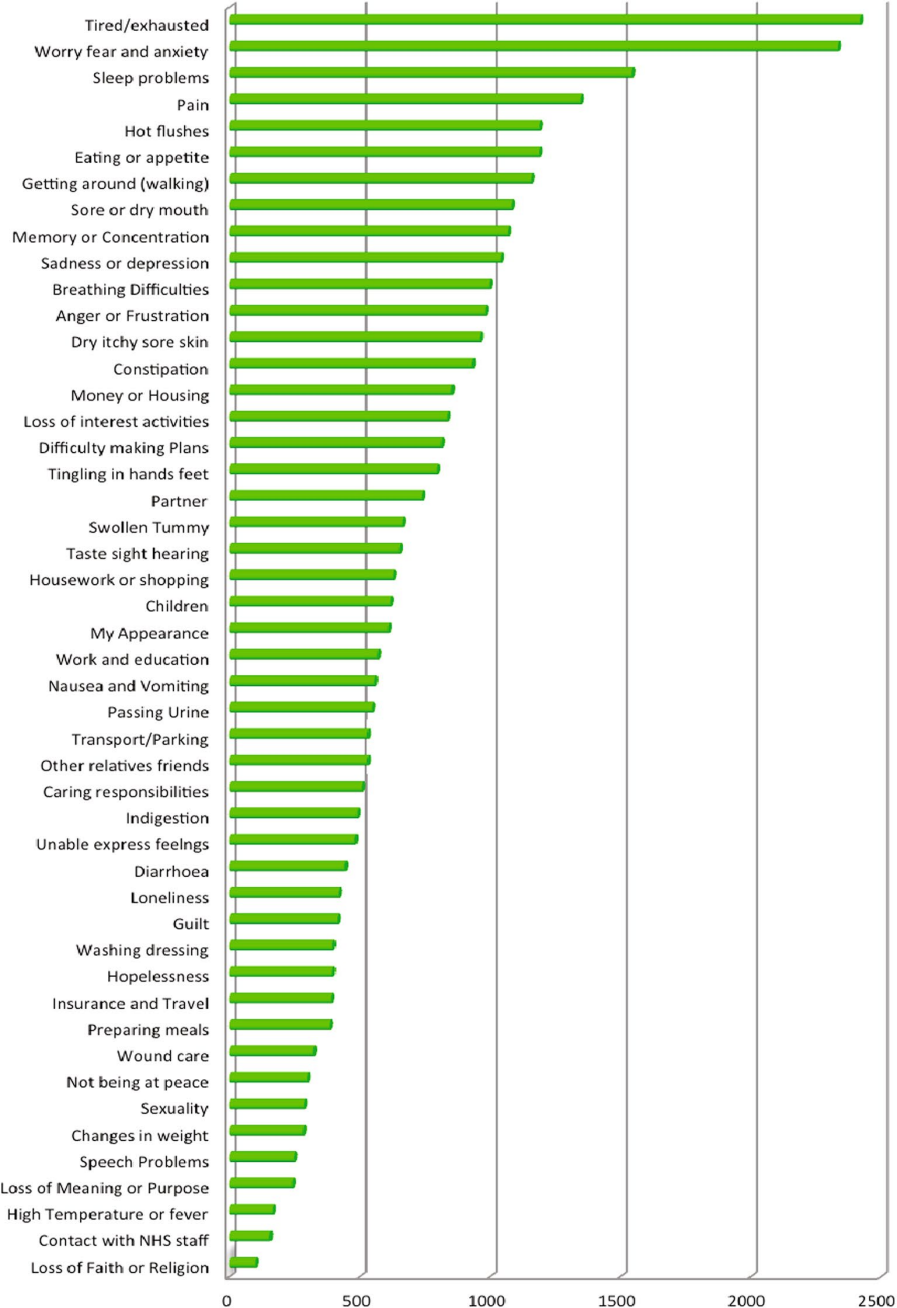
Problems ranked by frequency

The majority of the sample was classified as newly diagnosed (n = 1860) with 1259 described as on follow up, 1212 on treatment, 935 end of treatment and 155 not specified. In terms of treatment status 3675 were described as curative, with 1746 variously categorized as having end of life, palliative or life prolonging treatment. Seventy different diagnostic categories were identified. Figure 1 illustrates all the diagnostic categories with more than 20 participants in each. Figure 2 shows the frequency of each of the 48 problems on the eHNA.

## Analytic plan

The first objective was undertaken using Rasch analysis. The second used principal component analysis. This section details the assumptions, terminology and techniques associated with each objective.

*Objective 1* Test the degree to which the eHNA measures holistic concerns.

Rasch analysis begins with the assumption that the questionnaire items (in this case each questionnaire item is a ‘concern’) measure a *single latent trait* and that there is a hierarchy of responses, meaning higher items are likely to be endorsed only by those with higher levels of concern. Rasch analysis envisages a particular relationship between a participant’s score on an item and their position along the latent trait (Watson et al. 2011) and uses an iterative algorithm to test the data obtained against these expectations. The key output of this analysis relevant to objective 1 is ‘*item location*’, ‘*unidimensionality*’ and ‘*item invariance*’.

*Item location* is an expression of the likelihood of positively endorsing a particular item. Rasch analysis places all the items on a continuum, where the items most likely to be positively endorsed are at the bottom and the items least likely to be endorsed are at the top of this continuum. When using Rasch analysis to test for a scale measure each item should ideally contribute essential and preferably unique information in a hierarchical manner. However, the purpose of the eHNA is *not* to measure a *scale* of concerns but rather to capture holistic needs. To this extent a more logical outcome would be a *small spread* of location. A small spread of item location would show that the items are equivalently important (concerning) to people in this sample, which is what this particular Rasch analysis would hope to show.

In order to check whether all the items measure something of the same trait Rasch analysis also tests for *unidimensionality*. This test checks whether the data form a single factor. In other words it tests that the questionnaire is only measuring one latent trait (concerns), as opposed to measuring other variables. This is achieved by calculating ‘item fit’ as measured using the mean-square residual fit statistic (MSR). The ideal value is 1, but variation from 0.7 to 1.3 would indicate acceptable fit to the Rasch model in a sample this size (Bond and Fox 2007).

The final check for *item invariance* examines whether some groups of people (e.g. males/females) are responding differently (Teresi and Fleishman 2007). Item invariance is assessed here by the (DIF) statistic using the Mantel–Haenszel (MH) approach (Linacre 2015a). Ideally DIF would be minimal, although some could be expected. For example it could be predicted that females may respond differently to presence of ‘hot flushes’ for example. Testing for DIF according to gender will show if and how this is the case in this dataset and would further show if any other items may display differences according to gender. Likewise it may be the case that people who are categorized as being curative may respond differently to those classified as non-curative patients. Again, differential item functioning can test this. The Rasch Rating Scale Model (Bond and Fox 2007) was used to examine item location, item fit and item invariance (Williams et al. 2012) in WINSTEPS (version 3.81.0).

*Objective 2* Assess the factor structure of the eHNA.

Unlike Rasch analysis, factor analysis assumes all items are equally likely to be endorsed by respondents. It then examines covariance in response patterns in order to infer factors responsible for the covariance. Groups of variables that correlate closely enough to each other are considered to be unobserved factors (Dancey and Reidy 2014). Factor structures are useful in psychometrics as they help identify elements of a construct that may be clinically relevant. For example Petrides’ measure of emotional intelligence entails four elements: sociability, well-being, self control and emotionality. Scoring high or low on a particular factor could be useful to know so as to link in with the literature on sociability, well-being and so on. However, the purpose of the eHNA is *not* to point to general factors but to identify individual needs. An ideal factor analysis in this case would therefore result in 48 factors of equivalent strength, one for each item.

Factorability tests were first run on the data to ensure sampling adequacy. Principal component analysis (PCA) was then used to calculate a matrix of correlation coefficients drawn from the 48 measured variables (Clark-Carter 2010). Pearson’s correlation coefficient is used within PCA to construct correlation coefficients within the −1 to 0 to +1 range. Factor loadings describe the correlation of an individual item to an identified factor. PCA provides a table of the simplest factor loadings by tabulating each variable’s factor loading on each factor identified (Brace et al. 2009). The Kaiser criteria using eigenvalues greater than 1 was used to determine whether the item was retained or not (Lund and Lund 2015). Based on the assumption that the 48 items on the eHNA would likely be correlated, oblique techniques were used to better recognise potential association between variables (Brace et al. 2009). In summary, the PCA used direct oblimin rotation with Kaiser normalisation.

## Results

### Rasch analysis

Table 1 shows how responses fit the Rasch model. It shows that there is very little difference in location between the items. A good spread of items in a scale would hope to cover 4 logits (Linacre 2015b). These 48 items all fit within 0.69 logits, suggesting that there is very little to differentiate them in terms of whether one item is more ‘concerning’ than another. This fits very well with the concept of eHNA as a method of identifying holistic needs. Also, all but one of the items are a fit to the Rasch model in terms of dimensionality because they have an infit mean square of less than 1.3 and more than 0.7. All the items fit with the exception of item 21. This item, ‘*loss of faith or other spiritual concern*’ warrants further psychometric investigation, as it is unusual in not fitting. Misfit could be an artifact of being the least checked item (only 101 people checked this item in total and therefore some response categories had lower than ideal numbers for optimal results from the Rasch analysis). With this caveat, the above tests show that the eHNA measures a coherent construct (concerns) and consists of items that are equivalent in their importance to people.

**Table 1:** Item location, fit, standard error and DIF by gender and curative

Entry	Item location	Infit msq	SE	Items	DIF: gender	DIF: curative
1	−0.11	0.93	0.01	Anger or frustration		
2	−0.1	1.03	0.01	Breathing difficulties		×
3	0.01	1.17	0.01	Caring responsibilities		
4	0.14	1.3	0.01	Changes in weight		
5	−0.04	1.2	0.01	Children		
6	−0.06	1	0.01	Constipation		×
7	0.3	1.26	0.02	Contact/communication…NHS staff		
8	0.1	1.08	0.01	Diarrhoea		
9	−0.08	0.96	0.01	Difficulty making plans		
10	−0.07	1.04	0.01	Dry, itchy or sore skin		
11	−0.14	0.94	0.01	Eating or appetite		×
12	−0.16	1.05	0.01	Getting around (walking)		×
13	0.06	1.06	0.01	Guilt		
14	0.28	1.26	0.02	High temperature or fever		
15	0.06	1.07	0.01	Hopelessness		
16	−0.12	1.04	0.01	Hot flushes/sweating	×	×
17	−0.02	0.94	0.01	Housework or shopping		
18	0.08	1	0.01	Indigestion		
19	0.09	1.16	0.01	Insurance and travel		
20	0.06	1.07	0.01	Loneliness or isolation		
21	0.36	1.41	0.02	Loss of faith or…spiritual concerns		
22	−0.08	0.85	0.01	Loss of interest/activities		
23	0.18	1.13	0.01	Loss of meaning or purpose of life		
24	−0.11	0.86	0.01	Memory or concentration		
25	−0.11	1.15	0.01	Money or housing		
26	−0.01	1.07	0.01	My appearance		
27	0.04	1.04	0.01	Nausea or vomiting		×
28	0.13	1.13	0.01	Not being at peace … the past		
29	0.01	1.16	0.01	Other relatives/friends		×
30	−0.18	1.03	0.01	Pain		×
31	−0.08	1.14	0.01	Partner		
32	0.02	1.24	0.01	Passing urine		
33	0.1	0.96	0.01	Preparing meals/drinks		×
34	−0.13	0.87	0.01	Sadness or depression		
35	0.13	1.28	0.01	Sexuality		×
36	−0.2	0.91	0.01	Sleep problems/nightmares		×
37	−0.1	0.99	0.01	Sore or dry mouth		
38	0.21	1.14	0.01	Speech problems		
39	−0.03	1.15	0.01	Swollen tummy or limb		×
40	0	0.97	0.01	Taste/sight/hearing		×
41	−0.03	1.07	0.01	Tingling in hands/feet		
42	−0.32	0.74	0.01	Tired/exhausted or fatigued		
43	0.01	1.21	0.01	Transport or parking		
44	0.03	1	0.01	Unable to express feelings		
45	0.09	1.02	0.01	Washing and dressing		×
46	−0.01	1.15	0.01	Work and education		×
47	−0.33	1	0.01	Worry, fear or anxiety	×	×
48	0.14	1.21	0.01	Wound care after surgery		×

Some of the variance in the responses was a product of differential item functioning (DIF), indicated as either present or not in Table 1 according to gender and whether people were categorized as curative or not. It is important to keep in mind that DIF is not problematic in itself but rather helps better understand the construct under study (Linacre 2015b). It is helpful conceptually here as the items 16 and 48 demonstrate DIF in the same direction in relation to gender. Whilst the item on hot flushes is predictable as mentioned earlier, the DIF according to worry suggests that women appear to worry *differently* to men. Further, note that a number of items are answered differently by people who are curative. This will be returned to in the discussion.

### Factor analysis

Two factorability tests of the data were performed to analyse for sampling adequacy prior to factor extraction:

1. The Kaiser–Meyer–Olkin (KMO) test identifies the amount of variance within the data that can be explained by any unforeseen factors. Values between 0.5 and 1.0 are considered acceptable (Brace et al. 2009). The results of the KMO test showed an index of 0.917, which falls towards the preferred higher values closer to 1.0.
2. The Bartlett test of sphericity (BTS) results show a highly significant finding from the BTS of (χ^2^ = 36111.980, df = 1128, *p* < 0.0001).

The results of both tests confirmed that the data satisfied psychometric criteria for principal component analysis (PCA) to be undertaken.

The PCA showed 46.084 % of the cumulative variance was explained by 12 factors with eigenvalues greater than 1. The analysis therefore generated a 12 factor solution. The pattern matrix (Table 2) shows the regression co-efficients on each of the 48 items from the eHNA. The 48 items are listed on the first column and the 12 factors are the remaining columns (listed 1–12). Regression coefficients smaller than 0.1 have been omitted for ease of interpretation. The highest value regression coefficients are highlighted to show the most important items associated with each factor (Field 2013). For example factor 5 consists of two items: ‘contact/communication with NHS staff’ (loading coefficient 0.529) and ‘wound surgery’ (loading coefficient 0.611). Other items loading on to factor 5 show a higher coefficient in relation to a other factors and therefore load onto the factor for which their loading coefficient is the highest. For example in factor 5 the item ‘loss of faith or spiritual concerns’ shows a factor loading of 0.33, which is reasonable high. However, it has its highest loading against factor 1 (0.52), and so has a stronger association with factor 1. It should be noted that ‘cross-loading’ makes PCA difficult to interpret cleanly, although values around 0.3 are not considered too problematic, especially when there is a much larger loading on another factor.

**Table 2:** Pattern matrix

	Item	Pattern matrix factor											
		1	2	3	4	5	6	7	8	9	10	11	12
1	Anger or frustration	*.420*	.182		.131		.155						
2	Breathing difficulties			.124		−.125		*.251*		.156	.156	.250	.160
3	Caring responsibilities			.193	*.685*								
4	Changes in weight						−.112			.157			*.796*
5	Children				*.713*								
6	Constipation							.*511*	.213		.102		
7	Contact/communication with NHS staff		.145		.114	*.529*		.175					
8	Diarrhoea								*.253*	.237	.130		−.230
9	Difficulty making plans	*.400*	.117		.162	−.154						−.200	−.182
10	Dry, itchy or sore skin									.597			
11	Eating or appetite		.344	.120				.124	*.488*	−.140			.149
12	Getting around (walking)			*.406*		−.186		.378		.210		.109	.119
13	Guilt	*.454*			.277								
14	High temperature or fever		−.174			.134	*.532*	−.171	.289	.123			−.140
15	Hopelessness	*.587*							.130				
16	Hot flushes/sweating						*.728*						
17	Housework or shopping			*.747*									
18	Indigestion		−.144					.155	*.466*				
19	Insurance and travel			.113		−.124	−.270	−.123		.194	*.482*	−.305	−.166
20	Loneliness or isolation	*.548*		.129									.101
21	Loss of faith or other spiritual concerns	*.520*	−.181			.330				.140		.156	
22	Loss of interest/activities	*.506*	.144	.158		−.198						−.135	
23	Loss of meaning or purpose in life	*.678*				.150							
24	Memory or concentration	.186	*.253*	.150		−.109	.231		−.155	.116		−.166	
25	Money or housing				.176		.144				*.628*	.116	
26	My appearance			.154		.146	.182					*−.398*	.268
27	Nausea or vomiting		.118						*.714*				
28	Not being at peace/regret about past	*.568*	−.101										
29	Other relatives/friends				.546								
30	Pain		.142					*.485*				.134	
31	Partner				.656								
32	Passing urine							*.625*	−.158			−.333	−.225
33	Preparing meals/drinks			.*812*									
34	Sadness or depression	.*627*				−.115							
35	Sexuality							.160				*−.696*	
36	Sleep problems/nightmares		.207				*.523*	.129	−.156		.142		
37	Sore or dry mouth		*.511*					.151	.178	.150		.134	
38	Speech problems		*.609*	.110		.228		−.102					
39	Swollen tummy or limb		−.195					*.504*	.186				.177
40	Taste/sight/hearing		*.491*						.257	.262			
41	Tingling in hands/feet					−.117				.*665*		−.111	
42	Tired/exhausted or fatigued	.118	.200	.109		−.171	*.309*	.194	.115	.136	.108		
43	Transport or parking		.127	.126				.113	−.110	−.115	.557		
44	Unable to express feelings	*.496*	.169		.102	−.121							
45	Washing and dressing			.*762*									
46	Work and education		−.135				.101				.596	−.206	.147
47	Worry, fear or anxiety	*.338*	.142		.222	−.126	.112			−.270		−.114	
48	Wound after surgery		.134			.611							

The italised cells pick out the items most strongly correlated with the identified factor

Lund and Lund (2015) suggest that as a rule of thumb a factor structure should be retained if it accounts for at least 60–70 % variance in total. Alternatively each retained factor should explain at least 5 % variance. The more variance explained the more meaningful are the factors that have been identified. Table 3 shows that factor 1 consisted of 11 correlated items: Anger or frustration, difficulty making plans, guilt, hopelessness, loneliness or isolation, loss of faith or other spiritual concerns, loss of interest/activities, loss of meaning or purpose in life, sadness or depression, worry, fear or anxiety. This emotional/spiritual factor accounted for the largest amount of variance (15.574 %). Factor 2 consisted of 4 correlated items accounting for 5.097 % of the variance (memory and concentration, sore or dry mouth, speech problems, taste/sight/hearing). The factor therefore mainly pertained to oral or sensory issues, although the inclusion of the weakly correlating item (0.253) ‘memory and concentration’ makes straightforward interpretation difficult. Factor 3 entailed four correlated items related to ‘activities of daily living’ but only accounted for a further 3.360 % of the variance. The rest of the factors each explained even less variance.

**Table 3:** Variance explained and reliability analysis (Cronbach’s alpha) for each of the 12 factors

Factor	Items	Reliability analysis	% Variance explained	Cumulative % variance explained
1 Emotional/spiritual	Anger or frustration, difficulty making plans, guilt, hopelessness, loneliness or isolation, loss of faith or other spiritual concerns, loss of interest/activities, loss of meaning or purpose in life, sadness or depression, worry, fear or anxiety	α = 0.784	15.574	15.574
2 Oral effects^a^	Memory and concentration^a^, sore or dry mouth, speech problems, taste/sight/hearing	α = 0.459	5.097	20.671
3 Activities of daily living	Getting around, housework or shopping, preparing meals or drinks, washing and dressing	α = 0.687	3.360	24.031
4 Caring/relationships	Caring responsibilities, children, other relatives/friends, partner	α = 0.610	2.955	26.986
5 Surgery	Contact/communication with NHS staff, wound care after surgery	α = 0.199	2.732	29.718
6 Physiological effects	High temperature or fever, hot flushes/sweating, sleep problems/nightmares, tired/exhausted or fatigued	α = 0.480	2.595	32.313
7 Toilet/pain	Breathing difficulties, Constipation, pain, passing urine, swollen tummy or limb	α = 0.456	2.524	34.837
8 Gut	Diarrhoea, eating or appetite, indigestion, nausea or vomiting	α = 0.450	2.372	37.209
9 Treatment outcomes	Dry, itchy or sore skin, tingling in hands/feet	α = 0.273	2.303	39.512
10 Practical	Insurance and travel, money or housing, transport or parking, work and education	α = 0.479	2.234	41.746
11 Sexuality	My appearance, sexuality	α = 0.317	2.222	43.968
12 Weight	Changes in weight	One item only	2.166	46.084

^a^Memory and concentration are clearly conceptually unrelated to the other three items pertaining to oral effects. The categorization of this factor as oral effects therefore only represents conceptual commonalities between three of the four items

In other words, adopting Lund & Lund’s criteria for retaining factors, only factors 1 and 2 could be considered for retention as they each explained more than 5 % variance each. However, even though factor 2 explained slightly more than 5 % variance (5.097 %) it was difficult to interpret as discussed above. Further, the 12-factor solution only explained 46 % variance, somewhat short of the lower 60 % benchmark for acceptable fit.

In summary then, with the exception of the emotional/spiritual factor (Factor 1) it appeared that the eHNA was largely unfactorable. In order to further examine this conclusion the eHNA and its potential factors were assessed for internal consistency. The internal consistency of a measure consisting of all 48 items eHNA was: α = 0.874. This supports the conclusions of the Rasch analysis that the eHNA as a whole is a reliable and consistent measure of concerns. In order to examine the reliability of the other factors they were examined individually. The results of this analysis are in Table 3 and show that for the emotional/spiritual factor the internal consistency was good, with a solution of α = 0.784. The other subscales did not reach this level of reliability suggesting that they have less than acceptable internal consistency for clinical use. This further supported the preliminary conclusion that with the possible exception of factor 1 the eHNA was unfactorable in this sample.

## Discussion

Rasch analysis is usually used to test how well a particular measure operates as a scale measure. Here it has been used to test the opposite. That is, this measure of holism should ideally *not* operate as a scale because all the items should be as important as each other. Interpreting the Rasch analysis measures of separation in this case is therefore unusual. The range of the items is only 0.69 logits. Any value under 4 is usually considered problematic (Linacre 2015a) in scale development. In this case however the results are conceptually coherent with the attempt to ascertain holistic needs by showing that all items are equivalently important. This element of the Rasch analysis shows the results of the assessment to be conceptually coherent with its purpose.

However, this does not mean that the individual concerns that make up the eHNA are equally important for everybody. Recall that analysis of differential item functioning showed that people responded to some items differently according to gender, or whether they were classified as curative or not. An example of what this translates to in general clinical terms is given in Table 4. It shows that people categorized as curative spent a shorter period of time on average completing HNA compared to those not curative. They also recorded fewer problems on average. An independent-samples *t* test was run to determine if these differences were statistically significant.

**Table 4:** Mean scores on key variables according to whether curative or not

	Curative or not	N	Mean	Std. deviation	Std. error mean
Group statistics					
Total HNA score	Curative	3671	26.08	34.312	.566
	Not curative	1293	41.13	41.040	1.141
Number of problems	Curative	3675	5.86	5.460	.090
	Not curative	1297	7.64	6.576	.183
Minutes and seconds for HNA completion	Curative	3675	6 min 56 s	5 min 53 s	6 s
	Not curative	1297	8 min 36 s	7 min 5 s	11 s

The eHNA took less time to complete for those deemed curative (*M* = 6 min 56 s, *SD* = 5 min 50 s) than those deemed non curative (*M* = 8 min 37 s, *SD* = 7 min), a statistically significant difference, *M* = 1 min 37 s, 95 % CI (1 min 14 s, 2 min 6 s), *t*(1962) = −7.6, *p* < 0.001. Those deemed curative also reported less problems on average (*M* = 5.86, *SD* = 5.46) than those deemed non curative (*M* = 7.64, *SD* = 6.58), a statistically significant difference, *M* = −1.78, 95 % CI (−2.12, −1.38), *t*(1962) = −8.74, *p* < 0.001.

As well as being clinically useful information this finding provides further evidence that eHNA is conceptually coherent with its purpose in practice. Those not curative could be predicted to be likely to have more complex concerns and may also be suffering the effects of treatment more (Baile et al. 2011). What these results show is that clinicians are giving more time to those with greater self-identified need. It also means that for planning purposes clinicians can be aware of the additional time required and the likely enhanced distress in order to plan and respond accordingly.

**Fig. 3:**
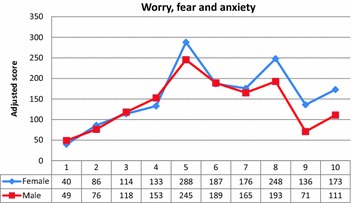
Comparison of males and females on score for ‘worry, fear and anxiety’. Male scores have been multiplied by the ratio of total females/total males to make the comparison equivalent on *y axis*. The numerical scale on *x axis* (1–10) is the distress score recorded by each respondent, with 10 being maximum

What was perhaps more surprising in the Rasch analysis (Table 1) was the differential item functioning apparent in relation to gender and ‘worry, fear and anxiety’. This was the only item apart from ‘hot flushes and sweating’ that displayed a different response patterns according to gender. Further investigation showed that females scored more often at the higher end of this item. Figure 3 shows that at the lower end of the scores for worry both males and females scored equivalently, but the response patterns diverge at five (the mode) and at eight and over, with females demonstrating more moderate and severe levels of worry, fear and anxiety than males overall. It might therefore be prudent for clinicians to explore the meaning of this item in both genders in more detail if checked, as it is unclear from this analysis whether females are more worried, males are more reticent to check the higher scores, or some other explanation exists.

In summary the Rasch analysis showed that the eHNA is a valid measure of holistic concerns. Further it showed that all 48 concerns were equally ‘concerning’. This meant all items should be retained within the checklist according to this analysis unless there is good clinical reason to remove any. The item about spiritual concerns was the only item that was a doubtful fit to the model, and therefore administrators should be aware that responses to this item might need further clarification/exploration over and above the usual support offered. From a psychometric perspective, this item was the least answered of all the items and there is therefore the chance that misfit may simply be an artifact of sampling error.

In relation to the PCA, given that the best fitting factor solution returned 12 factors and this solution only accounted for 46 % variance it could be argued that the eHNA is non factorable. This would also fit with the conclusion that the eHNA is best considered as a tool to identify ‘holistic’ concerns. That is, each individual item on the checklist may not contribute much information to any underlying factor score, but rather each item is instead most important as a stand-alone piece of information. In other words reducing the item set to a smaller set of factors is again philosophically inconsistent with the concept of ‘holism’ underpinning this measure.

The only possible exception to this conclusion was the first factor that showed good internal reliability (0.784) and a strong association with eHNA total score (0.817). The clinical benefit and utility of this factor remains to be seen, but perhaps it could be useful where the patient checks a large number of problems. It could help in this case reduce an ostensibly unmanageable set of individual problems into coherent action. The purpose of understanding a factor in this case would be to better target support. However, because the mean number of problems identified was just over six it is unlikely that understanding a factor structure would help in these cases, given that helping people with their *individual* problems would be the most coherent action of the clinician. The only time a factor interpretation may be useful would be if people ticked a large number of problems, where understanding any underlying factor structure may be helpful in prioritizing actions. To this end it would be worth conducting further confirmatory tests on this dataset to better understand the hypothesized factor structure obtained here.

### Limitations

Despite the large dataset a limitation of the sample is that there was no information on the number of people who completed a holistic needs assessment and did *not* raise any concerns. There was no data on ethnicity and it is known that some ethnic groups have been shown to report different scoring patterns in other comparable cancer quality of life measures (Pagano and Gotay 2005). We have also not had space to explore the outcomes of eHNA (i.e. actions taken as a consequence of completing the eHNA) in this dataset. Further investigation is being planned.

## Conclusion

The aim of this study was to evaluate the psychometric properties of the eHNA by examining its construct validity. The Rasch analysis showed that the 48 items measured a single latent trait. It showed that all items were equivalently important and that no items should be removed from the checklist on the basis of fit. The principal component analysis supported this conclusion to the extent that no factor explained enough variance to warrant reduction in the item bank. Reliability analyses confirmed the best fit included all the items. The eHNA is a valid and reliable assessment of holistic needs.
